# The Pew System

**Published:** 1861-08

**Authors:** 


### THE PEW SYSTEM

Has its advantages and disadvantages, and there is reason to believe that, under the present order of things, it is proper and necessary that families should rent or own their pews, with modifications according to circumstances. It might answer an excellent practical purpose for each pew owner to open the door (if there is a vacancy) as soon as he knows that all the members of the family to be expected on the occasion are in. Such an arrangement would relieve the sexton of a delicate duty, and would be a tacit invitation to those waiting for a seat at the threshold, and thus enable all to be accommodated within a few minutes of the commencement of the services.

The Saturday newspapers regularly contain a column or more of notices of public service at particular places, with a direct or implied invitation to the public to attend; and yet, in too many cases, when the public do attend, they are negligently, if not discourteously and insultingly treated. The daily press contained a notice lately, that on the last Sabbath of the month, the house on Fourth Avenue where Dr. Bellows officiates, would be re-opened, when that gentleman would address the people. For a variety of reasons we attended with two ladies, one of them of great culture from a distant State. Having a special desire to hear the distinguished speaker, we were there some minutes before the time. There was quite a crowd of persons at the vestibule; perhaps a hundred were already seated. There was one sexton to show strangers to seats, but this was so slow a process that the crowd was steadily increasing. Those who were entitled to seats could scarce pass, and the impatient exclamation from a richly-dressed woman—"let me by"—was no more agreeable to hear than the voice of the sexton, which soon followed; for, seeing that with all his efforts there was no visible diminution of the number of those who were waiting, he made a kind of speech to the crowd, which, with a sweeping wave of the hand, seemed to mean that all were included, "there are plenty of good seats in the gallery;" but whether for ladies, gentlemen, or pigs, was not stated. We concluded, under all the circumstances of the case, to withdraw to some more hospitable clime, and passed on to Calvary church, where the model sermonizer officiates. As no man can listen to Dr. Hawks five minutes without profit and instruction, we determined to make some sacrifices, feeling sure, from very many previous occasions, that we would be fully compensated. We took the liberty of finding seats for the ladies at once, and retired among the undistinguishable crowd of waiters, to bide our time. A number of pews had but a single occupant. They were coming to the inimitable litany, and we had no seat. A lady in front of us had several times looked around, as if she were distressed at seeing so many persons standing so long, and taking it as an invitation, we made ourselves the second occupant of her pew; and as she took some pains to show us all the places, and with great courtesy invited us nearer to her so that we could see and listen to the greatest advantage, we soon felt at home; but at the commencement of the discourse there were ladies and gentlemen still standing. At the end of the service we paid for our seat in the way of a general collection. At the opera we pay first, and are promptly and deferentially conducted to a seat, without the penance of standing twenty minutes or half an hour. These things ought not to be. Considerate persons will wait patiently for many minutes, but the great majority are so ruffled in their minds if there is a longer waiting than five or ten minutes, that they are not benefited by anything that follows. It is therefore suggested,

That one or two persons be stationed at the head of each aisle, from fifteen minutes before the service until the commencement of the second hymn, as at Mr. Chapin's, in Broadway, thus showing that his Gospel is literally for all, and not for those only who can purchase pews at one and two thousand dollars apiece.

